# The Prolactin Gene: A Paradigm of Tissue-Specific Gene Regulation with Complex Temporal Transcription Dynamics

**DOI:** 10.1111/j.1365-2826.2012.02310.x

**Published:** 2012-07

**Authors:** K Featherstone, M R H White, J R E Davis

**Affiliations:** * Developmental Biomedicine Research Group, Faculty of Medical and Human Sciences, University of ManchesterManchester, UK; † Systems Biology Centre, Faculty of Life Sciences, University of ManchesterManchester, UK.

**Keywords:** pituitary, prolactin, transcription, dynamics, chromatin

## Abstract

Transcription of numerous mammalian genes is highly pulsatile, with bursts of expression occurring with variable duration and frequency. The presence of this stochastic or ‘noisy’ expression pattern has been relatively unexplored in tissue systems. The prolactin gene provides a model of tissue-specific gene regulation resulting in pulsatile transcription dynamics in both cell lines and endocrine tissues. In most cell culture models, prolactin transcription appears to be highly variable between cells, with differences in transcription pulse duration and frequency. This apparently stochastic transcription is constrained by a transcriptional refractory period, which may be related to cycles of chromatin remodelling. We propose that prolactin transcription dynamics result from the summation of oscillatory cellular inputs and by regulation through chromatin remodelling cycles. Observations of transcription dynamics in cells within pituitary tissue show reduced transcriptional heterogeneity and can be grouped into a small number of distinct patterns. Thus, it appears that the tissue environment is able to reduce transcriptional noise to enable coordinated tissue responses to environmental change. We review the current knowledge on the complex tissue-specific regulation of the prolactin gene in pituitary and extra-pituitary sites, highlighting differences between humans and rodent experimental animal models. Within this context, we describe the transcription dynamics of prolactin gene expression and how this may relate to specific processes occurring within the cell.

## Introduction

The temporal dynamics of biological systems is a key component of tissue or organism function. In endocrine tissues, frequency encoding of information via pulsatile hormone secretion has long been appreciated to be important in the regulation of target systems (1). More recently, numerous genes have been shown to be transcribed in a temporal manner, resulting in stochastic pulses or bursts of transcription, including hormone-encoding genes (2,3). In single cell organisms, pulsatile gene expression has been hypothesised to increase the overall fitness of the population to changing environmental conditions (2). In multicellular organisms, the physiological relevance of pulsatile transcription is less clear, although links to cell lineage decisions have been made (4,5). More generally, pulsatile transcription may influence whole tissue responses by limiting the number of cells able to mount a transcriptional response at any given time. Overall pulsatile transcription may reflect the summation of a number of temporal processes occurring in the nucleus, including: on/off transcription factor dynamics, transcription complex assembly, polymerase recruitment and activation, chromatin remodelling cycles, and recruitment of DNA to transcriptionally competent areas of the nucleus (transcription factories). In this review, we describe the transcription dynamics of the prolactin gene, which is used as a model of tissue-specific gene regulation, in the context of established mechanisms of prolactin gene regulation.

**Fig. 1:**
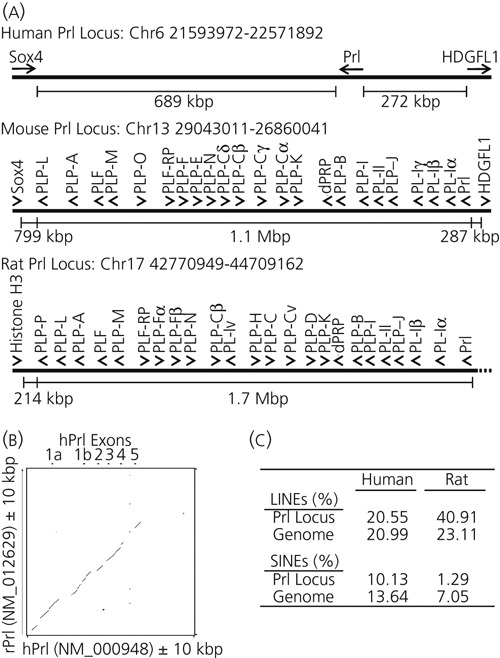
The prolactin gene locus. (a) The location and organisation of the human, mouse and rat prolactin loci (not to scale). The loci are orientated by the prolactin gene and this is reflected by the numbering of the locus position on the chromosome. The diagram illustrates the vastly different structure of the prolactin loci between primate and rodent species. Data from ensembl (human: release GRCh37, rat: release RGSC3.4, mouse: release NCBIM37) and (6,7). HDGFL1, hepatoma-derived growth factor like 1. (b) Alignment of the repeatmasked human prolactin gene ± 10 kbp sequence to the rat prolactin gene ± 10 kbp sequence shows sequence homology in noncoding sequences in upstream regulatory regions and in gene introns. Alignment generated using Pipmaker (8). (c) Repeat composition of the human and rat prolactin loci. The rat prolactin locus shows a substantial difference in repeat content in comparison with the genome average. Data derived from repeatmasker [A. F. A. Smit, R. Hubley and P. Green, unpublished data. Version: open-3.3.0 (RMLib: 20110419)] using ABBlast and default settings (10,11).

Prolactin is an important endocrine hormone, which is secreted primarily from lactotroph cells of the pituitary gland in a circadian manner, with increases in secretion occurring during the pro-oestrous phase of the rat 4-day oestrous cycle, as well as during pregnancy and stress. Prolactin in humans can also be generated locally in numerous tissues and cells, including the endometrium, brain, breast, skin, lymphocytes and adipocytes. Unsurprisingly, therefore, prolactin has been shown to function in numerous processes, including reproduction, metabolism, immunology and behaviour, as well as pathologically in cancer. The diverse expression of the human prolactin gene requires complex regulation, with the use of two independent promoters, which show differential responses to regulatory mediators and cell-type specific activity. This organisation of the human prolactin locus is not conserved across mammalian species and is markedly different from the rat and mouse prolactin loci, which have been used as experimental models of prolactin regulation for decades as a result of sequence conservation of important regulatory elements located proximally to the gene (Fig. 1a,b).

## Structure and regulation of the human prolactin locus

**Fig. 2:**
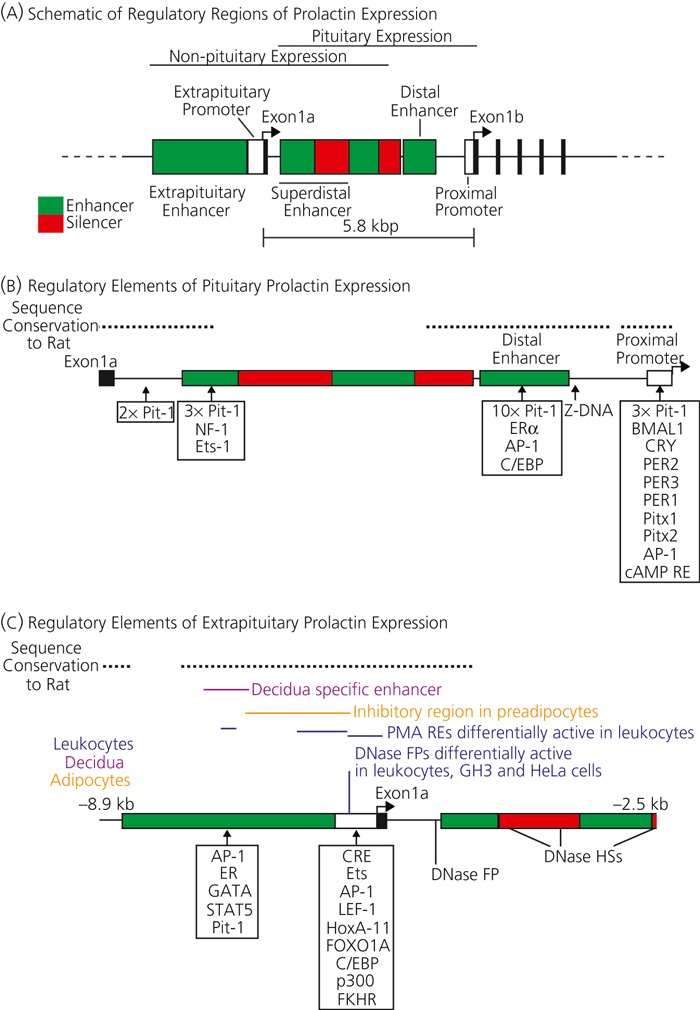
Schematic of regulatory elements in the human prolactin locus. (a) Organisation of regulatory elements in the prolactin locus. Exons (black boxes), promoters (white boxes), enhancers (green boxes) and silencers (red boxes) are shown. (b) Organisation of regulatory elements that facilitate expression in the pituitary. Transcription factors with demonstrated binding to the prolactin locus are listed. (c) Organisation of regulatory elements that facilitates expression in nonpituitary tissues via the upstream alternative promoter and exon1a. Elements with potentially specific activity within different extra-pituitary tissues are depicted above the locus and transcription factors with demonstrated binding to the locus are listed below. Numbering is from the start of exon 1b. HS, Hypersensitive site; FP, footprint; RE, response element.

In humans, the prolactin locus exists in a gene poor region of the genome and consists of a single gene containing five coding exons, transcribed directly from a pituitary specific promoter, and a noncoding exon transcribed from an alternative promoter, which drives expression in nonpituitary tissues. By contrast, in rodents, gene duplication has generated a large family of prolactin genes, at a single locus, with independent expression profiles and independent functions (9) (Fig. 1a). Sequence composition of the prolactin loci between these species is also distinct, revealed by the substantially altered proportions of different classes of interspersed repeats within the sequences (Fig. 1c). The lack of available human pituitary tissue and human-derived cell lines for experimental study has necessitated the use of experimental animal models for studies of prolactin function and gene regulation, with the rat being the animal model of choice for endocrinologists. The introduction of mouse gene knockout technology in 1989 resulted in increased studies in mice; however, the wealth of data in rats and recent reports of successful knockout of targeted genes in rats (12,13) could see rats continuing to be favoured for study in the future. New genomic technologies, as well as future developments, are also likely to affect experimental choices and may reduce animal use. For example, regulation of the human prolactin locus can now be studied using bacterial artificial chromosome (BAC) technologies, which has several advantages over the short DNA constructs used previously (14). These advantages include the ability to assess regulatory elements within their native context in combination with other regulatory elements within the locus, the ability to search for long-range distal regulatory elements, the maintenance of chromatin influence on the locus, and the insulation of position effects from the DNA insertion site. Disadvantages to the use of BAC transgenes include truncation of the BAC and variable integration (position and copy number) into the genome. The utility of BAC constructs has been demonstrated in rat pituitary cell lines where a BAC construct, containing a large proportion of the human prolactin locus (up to 168 kbp), showed greater transcriptional activation to several known regulators of prolactin expression compared to a short promoter construct (5 kbp), indicating that as yet unidentified distal regulatory elements are present in the human prolactin locus (15). Regulation of gene activity from a distance is not uncommon and is likely to have important consequences for gene activity, as illustrated by global analyses of DNA binding sites, which show that proteins often bind to sequences far from known genes. In particular, oestrogen receptor (ER)α, a key regulator of pituitary prolactin expression, only has 4% of its binding sites within promoter regions (defined as −800 bp to +200 bp from transcriptional start sites) (16). Additionally, long-range interactions between DNA sequences, in *cis* and in *trans*, indicate that long distance regulation is important and influences the global organisation of DNA within the nucleus (17). Despite questions remaining as to the long-range regulation of the human prolactin gene, several important regions have been defined upstream of the gene (Fig. 2).

### Regulation of pituitary prolactin expression

Functional domains that mediate pituitary prolactin expression have been identified in the rat and human prolactin loci. A proximal promoter and distal enhancer are present in both species and share sequence homology (18,19). In the human locus, an additional upstream regulatory element, the superdistal enhancer, has been identified, although the functional significance of this element has yet to be fully characterised (20). In the rat, the distal enhancer and proximal promoter have been shown to physically interact, generating a chromatin loop (21,22), although the context of this chromatin loop within the full locus, where multiple genes may initiate tissue-specific chromatin loops has yet to be determined. The spatial conformation of the human prolactin locus is unknown and may be tissue-specific and complex as a result of the presence of several enhancer and silencer domains. The formation of chromatin loops is likely to be plastic, as demonstrated by the stabilisation of the prolactin enhancer-promoter loop by oestrogen treatment (22), with consequences for transcription dynamics.

In both species, pituitary prolactin expression is dependent on the Pit-1 transcription factor, a member of the POU homeodomain protein family (23,24). Pit-1 activity is modulated in several ways. Pit-1 can homo- and hetero-dimerise to numerous different proteins with competition between protein-binding partners, modulating the activity of the protein complex. The formation of different synergistic protein interactions by Pit-1 may, in part, be instructed by the binding site sequence (25). Pit-1 activity is also modulated by post-translational modifications. Phosphorylation of Pit-1 differentially affects Pit-1 binding affinity to different Pit-1 binding sites, alters the protein conformation when bound to DNA, influences the interaction of Pit-1 with other proteins, and may affect protein stability (26–28). Pit-1 can also be acetylated, although the functional significance of this is unknown (29). Finally, different Pit-1 protein isoforms exist that affect the transactivation potential of the protein (30). Modulation of Pit-1 function can lead to diverse and context dependent activity, which may facilitate cell-specific function, enabling Pit-1 to limit prolactin expression to lactotroph cells, despite its expression in other pituitary cell types. The basis of cell-type restriction of hormone expression is incompletely understood at present, although proposed mechanisms include synergistic interactions between Pit-1 with other factors (e.g. thyroid hormone receptor and nuclear receptor corepressor in somatotrophs) to generate a cell-type specific code (31). The locus control region in the human growth hormone locus also contributes to cell lineage restriction of growth hormone expression (32) and cell-specific regulation of growth hormone expression via a ZEB1 and an LSD1 corepressor complex has also been suggested to play a role (33).

Numerous molecules have been identified that influence prolactin expression levels, mainly through extensive studies in rats. The hypothalamus imparts significant control on prolactin production and secretion through the action of several neuropeptides and neurotransmitters. Dopamine is one of the most important hypothalamic mediators, maintaining prolactin gene expression and protein secretion under tonic inhibition, with rhythmic dopamine release influencing daily prolactin surges (34). Prolactin expression is also regulated by steroids, hormones and cytokines produced throughout the body. Thus, the level of prolactin expression can be considered as a balance between inhibitory and stimulatory influences originating from numerous organ systems. The integration of these signals is achieved by several intracellular messengers, including the phosphatidylinositol pathway, which increases intracellular calcium levels and activates protein kinase C; cAMP, which activates protein kinase A; and the mitogen-activated protein pathway (19,34,35). Activation of protein kinase C and increases in intracellular calcium and cAMP stimulate human prolactin gene expression, suggesting that these pathways are also operative in humans (36).

Numerous protein-binding sites are involved in mediating changes in prolactin expression levels. Pit-1 binding sites are required for responses to calcium and cAMP signalling (37,38), although whether this is mediated by Pit-1 or other factors (e.g. prolactin regulatory element binding protein) remains under question (39,40). AP-1, Ets, Pitx factors and ER binding sites also mediate responses to transcriptional stimuli.

Differences between rats and humans exist in their response to regulators of prolactin expression, as exemplified by oestrogen, which has a major influence on rat prolactin expression but may have a more limited action in humans. Oestrogen causes large increases in rat prolactin expression through synergistic activity with Pit-1 (41,42). In humans, modest responses to oestrogen are detectable in cell line models (43) and changes in oestrogen levels in human physiology do not always equate to changes in prolactin expression levels (19). Overall, these data suggest that prolactin expression levels result from the integration of numerous intracellular signalling pathways via the modified action of a number of transcription factors.

### Regulation of extra-pituitary prolactin expression

The expansion of the rat and mouse prolactin gene family has enabled the adaptation of prolactin proteins to environmental challenges, the result of which is the expression of prolactin family proteins in decidua and trophoblast cells, indicating that fine-tuning of prolactin levels is beneficial during pregnancy. Important insights into the function of individual prolactin family member proteins have been gained by mouse mutagenesis studies and a role for these proteins in reproduction, angiogenesis, haematopoiesis and in the immune system has been described (9). In humans, the single prolactin gene does not allow for similar versatility; instead, humans and primates have gained an alternative (extra-pituitary) promoter that allows expression in several nonpituitary cells and tissues, including endometrium, breast, brain, skin, prostate, adipose and haematopoietic cells (19,44). Instances have also been reported where extra-pituitary expression is driven by the pituitary (exon 1b) promoter (45–48); however, whether this reflects the physiological activity of this promoter *in vivo* is unclear and requires further investigation. Data on the potential functional significance of locally-produced prolactin expression in nonpituitary tissues are summarised in Table 1.

**Table 1:** Sites and Function of Extra-Pituitary Prolactin Expression

Site of expression	Function
Myometrium and decidualised endometrium (49)	Reduced prolactin expression levels are associated with recurrent pregnancy loss and impaired decidual programming (50)
Immune system (15,51–55)	Prolactin may have an immunomodulatory role: influencing the differentiation of T and B cells; having a costimulatory activity on T and natural killer cells and increasing immunoglobulin production. A prolactin polymorphism has been associated with autoimmune disorders including systemic lupus erythematosus and rheumatoid arthritis (56)
B-lymphocytes	
T-Lymphocytes	
Thymocytes	
Bone marrow stromal cells	
Granulocytes	
Monocytes	
Macrophages	
Breast (57)	The primary function of prolactin is to enable mammary gland development and lactation. High-normal circulating prolactin levels have been associated with an increased risk of breast cancer in women. Rodent models of locally produced prolactin protein can induce breast and prostate tumours (58)
Glandular tissue	
Adipocytes	
Skin	Prolactin may have a role in psoriasis, a skin disorder involving cellular hyperproliferation and inflammation (59, 60)
Brain (61)	Investigated primarily in rodents, local and systemic prolactin may have roles in regulating maternal behaviour, in responses to stress, in neurogenesis and regulation of the hypothalamic-pituitary-adrenal axis (62)
Fat (63)	Decreased prolactin secretion from fat depots may be associated with obesity (64)

The diverse expression profile of the prolactin gene in nonpituitary sites suggests a complex system of regulation enabling cell-specific expression and response to differential regulatory mediators. The extra-pituitary promoter evolved from a long terminal repeat-like transposable element (65), which causes the inclusion of an untranslated exon into the prolactin transcript (66). Although there is sequence homology across the extra-pituitary promoter region between humans and rats, a functional promoter does not exist in the rat (67). The species specificity of the extra-pituitary promoter has therefore limited previous studies to human cell lines and isolated tissues. The recent generation of a transgenic rat containing the human prolactin locus now allows further detailed studies of the function of the extra-pituitary promoter in whole animal physiology along with pharmacological intervention *in vivo*. The applicability of this system has been demonstrated by the expression of prolactin in immune cells following lipopolysaccharide- or thioglycollate-induced peritoneal inflammation and may prove useful in determining new sites of human prolactin expression (15,55).

The extra-pituitary promoter has dissimilar activity to the pituitary promoter, with Pit-1 independent activity and responsiveness to different regulators of gene expression (52). Regulatory sequences mediating extra-pituitary expression are detailed in Fig. 2. The extra-pituitary promoter shows cell-specific activity with differences in responses to physiological regulators and with regulation mediated by different regions of the promoter. Early studies detected differences in the activity of the 3kbp upstream extra-pituitary promoter sequence in lymphoid and endometrial stromal cells (52) and a decidua-specific enhancer has been identified within this sequence (68). Leukaemic cell lines have been shown to respond differentially to cAMP, phorbol myristate acetate and ionomycin via different regions of the extra-pituitary promoter (69). Similarly, progesterone and insulin have cell type-specific effects on extra-pituitary prolactin expression (19,64). The kinetics of responses are also cell-type specific, with cAMP inducing a biphasic response in endometrial cells, whereas lymphoid cells show an immediate single phasic response (70). Further work is required to fully dissect the functional elements of the extra-pituitary promoter and to determine how cell specificity is imposed.

## Real-time transcription dynamics of the prolactin gene

In the past few decades, increasing attention has been given to the temporal aspects of gene transcription, providing important insights into gene regulation. It has become clear that transcription activity is stochastic and is affected by intrinsic and extrinsic noise such that substantial cellular heterogeneity exists within cell populations (71). As a result, data derived from single cell analyses are often the most informative. Technologies used to assess single cell transcription dynamics include transcript counting methodologies such as RNA fluorescent *in situ* hybridisation and single cell quantitative reverse transcriptase-polymerase chain reaction, which provides a snapshot of the transcription activity of a population of cells. Temporal data have been derived by the indirect labelling of transcripts, through the inclusion of a reporter gene sequence downstream of the promoter of interest, or the direct labelling of transcripts using the MS2 RNA tagging system, with time-lapse microscopy detecting changes in reporter gene levels. A comprehensive review of technologies available for single cell analyses and mathematical modelling of quantitative data has recently been published (72).

Analyses of transcription dynamics in simple single cell systems, such as bacteria and yeast, have highlighted the stochastic and pulsatile nature of transcription and the contributions to this through intrinsic and extrinsic noise (73,74). In mammalian cells, efforts to determine transcription dynamics, through RNA counting or reporter techniques, have shown that transcription occurs in highly variable bursting patterns, in most cases, and with gene-specific kinetics (2,3,75). Chromatin remodelling has been suggested to be highly influential in setting up this transcription activity (75). The prolactin gene provides an interesting model in which to study transcription dynamics as a result of its complex tissue-specific regulation and altered activity in different physiological states.

### Prolactin transcription dynamics in cell lines and primary cell cultures

The temporal activity of prolactin gene transcription has been studied via indirect measurement of transcript abundance using luciferase or destabilised green fluorescent protein (GFP) reporter genes. Early research used a construct containing 5 kbp of the human pituitary promoter sequence followed by the luciferase gene, which was transfected into the GH3 somatolactotroph cell line (76,77) or adenovirally transduced into primary pituitary cells from the Syrian hamster (78). An alternative approach, by an independent group, involved the use of a construct containing 2.5 kbp of the rat pituitary promoter sequence followed by the luciferase gene, which was microinjected into primary pituitary cells from lactating rats (79,80). In both cases, expression of the luciferase protein was monitored by microscopic imaging using photon counting charge coupled device cameras. Studies using these approaches showed that prolactin expression was pulsatile, with large fluctuations in expression levels and that heterogeneous responses occurred between cells in unstimulated and stimulated conditions (76,78,81,82). In primary lactotroph cells, differences in calcium responses and basal expression levels have been suggested to account for heterogeneous responses to stimuli (81–83) in systems where cellular heterogeneity may account for differences in transcriptional activity. By contrast, transcriptional heterogeneity in clonal cell lines cannot similarly be attributed to cellular heterogeneity. Studies have suggested that prolactin transcription pulses may arise as a result of the direct regulation of the gene by components of the circadian system (84,85) or via circadian recruitment of transcriptional repressor proteins and chromatin remodelling (86). However, circadian timing of prolactin transcriptional pulses remains controversial because single cells show variable transcriptional periodicity within an experimental system, with reported average transcription cycle durations of 11, 15, 24 and 48 h (77,82,87), where differences probably result from the experimental system used. The lack of circadian timing in pulsatile prolactin transcription suggests that other oscillatory mechanisms may also operate within the system. Oscillatory behaviour is present within many cellular systems and, cumulatively, it can be envisaged that this results in heterogeneous transcription dynamics, which may appear to be stochastic. Oscillatory activity shown to influence transcription dynamics ranges from cell signalling to transcription factor activity. Cell signalling can impinge on transcription factor function as demonstrated by the modulation of the nuclear-cytoplasmic shuttling dynamics of the transcription factor Crz1 by extracellular calcium (88) and through tumour necrosis factor (TNF)α oscillatory signalling on nuclear factor (NF)-κB nuclear-cytoplasmic shuttling dynamics (89). Oscillatory behaviour in transcription factor activity was shown to have a functional consequence for downstream transcriptional events in both of these systems. Chromatin remodelling has also been shown to have a cyclical component, which, along with cyclical transcription factor recruitment, resulted in periodic transcription in several gene loci or reporter systems (90–92), suggesting, ultimately, that dynamic transcriptional processes result from responses to nuclear protein function and the nuclear architecture (93). At the prolactin locus, the involvement of several intracellular signalling pathways and transcription factors in the regulation of the gene probably facilitates the appearance of stochastic transcription dynamics, although the influence individual components have on transcription dynamics is not known. It should be noted that the dynamics of the prolactin gene are not completely stochastic as a result of the presence of constraints on gene expression (e.g. a transcription refractory period, as described below). Oscillatory factors that may directly influence prolactin transcription dynamics include: cyclical recruitment of ER and oestrogen signalling (94), cell cycle dependent modification of Pit-1 activity (95), TNFα signalling with subsequent nuclear-cytoplasmic shuttling of NF-κB (96) and calcium signalling.

**Fig. 3:**
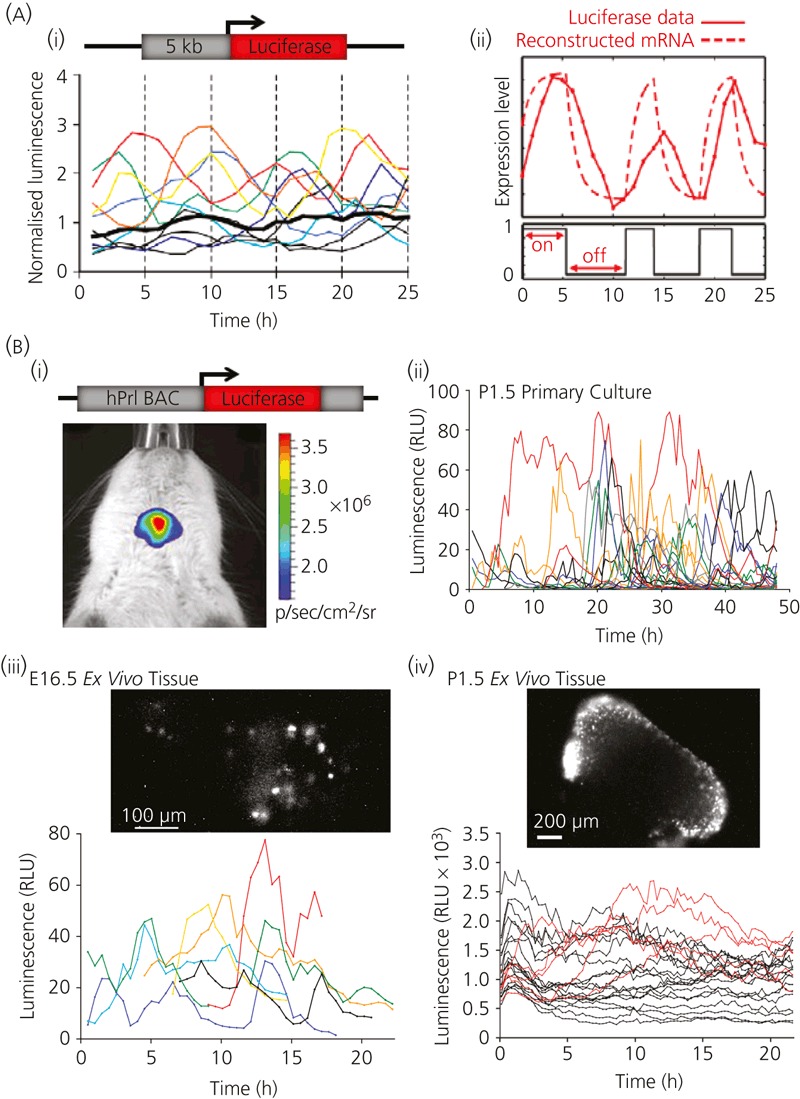
Transcription dynamics of the human prolactin gene. (a) Transcription activity of the human prolactin gene in the somatolactotroph GH3 cell line. Data are from Harper *et al*. (87). (i) Microscopic measurement of luciferase expression in individual GH3 cells shows that prolactin expression is highly dynamic with pulses of expression. (ii) Luciferase data were used to calculate mRNA expression dynamics of the prolactin gene by taking into account mRNA and protein degradation rates. These data were used to predict ‘on’ and ‘off’ transcription times using a binary switch model. A minimum ‘off’ time of approximately 3 h indicated the presence of a transcriptional refractory period. (b) A transgenic rat model with the luciferase gene controlled by the human prolactin locus has enabled investigations of transcription activity in pituitary tissue and primary cells. (i) *In vivo* imaging of luciferase expression in the pituitary using an IVIS Spectrum (Caliper Life Sciences, Hopkinton, MA, USA). Data from Semprini *et al*. (15). Technological developments may enable measurements of transcription activity *in vivo* in real-time in the future. (ii–iv) Luciferase expression reveals marked differences in transcription activity in dissociated cells (ii) and cells maintained within their native tissue environment (iv) and between cells from different stages of development (iii versus iv). Each trace in the graphs represents a single cell. Images of luciferase expression within pituitary tissue are shown above corresponding single cell luciferase expression data. Data from Featherstone *et al*. (103).

**Fig. 4:**
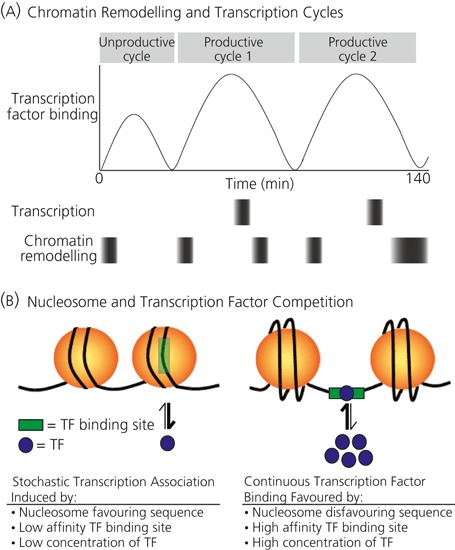
Chromatin influences on transcription dynamics. (a) Chromatin remodelling and transcription factor recruitment define cycles of transcription activity, demonstrated at the pS2 promoter. Active transcription was defined by the presence of phosphorylated RNA polymerase II. Adapted from Metivier *et al*. (98). (b) Nucleosome positioning is plastic and may compete with transcription factors for DNA occupancy affecting transcriptional stochasticity. Factors influencing the equilibrium between nucleosome and transcription factor occupancy include sequence preference for nucleosomes, the affinity of the transcription factor binding site and transcription factor concentration.

Detailed analysis and mathematical modelling of pulsatile prolactin transcription dynamics have recently been reported (Fig. 3a) (87). Independent reporter constructs were used to assess the level of correlation between two identically regulated transgenes at independent loci. The transcription profile of the transgenes was mathematically determined from the measured reporter protein levels and correlation analysis, using rank correlation coefficients, was used to assess the degree of coordination of transcription activity between the reporter genes. Transcription dynamics, in untreated conditions, were uncorrelated between the two reporters, indicating that prolactin transcription cycles and their timing are not a result of extrinsic factors such as cell cycle stage or circadian regulation but, instead, were inherent to the transcriptional process. Coordination of the independent reporter genes was achieved by modification to chromatin structure, through a histone deacetylase inhibitor (Trichostatin A), suggesting that chromatin-regulated cycles may determine transcription pulse activity. Stochastic binary switch modelling of the data to estimate the distribution of transcriptional ‘on’ and ‘off’ times found a minimum ‘off’ period of 3 h revealing the presence of a transcriptional refractive phase in the transcription cycle. Transcriptional refractory phases have also been detected by others using several different promoter sequences (3). Chromatin cycles in transcriptional processes have been identified at other genomic loci and may be complex (92,97,99). At the pS2 gene, transcriptionally unproductive histone modification cycles precede multiple transcriptionally productive cycles before returning to a basal state (Fig. 4a) (92,97). Whether functionally different chromatin cycles occur at the prolactin locus remains to be determined and awaits the full characterisation of chromatin structure during prolactin transcription.

### Prolactin transcription dynamics in pituitary tissue

With numerous studies investigating real-time transcriptional dynamics in cultured cells, particularly bacteria, yeast and, more recently, mammalian cell lines, there is now a need for the characterisation of real-time transcription dynamics in physiological processes in whole organ systems. The importance of characterising transcription dynamics *in vivo* in organ systems is highlighted by the different mobility of transcriptional components in cell lines and mammalian tissues (100). The recent development of transcription reporter systems in transgenic animals is starting to enable studies of transcription dynamics in intact tissue. A knock-in mouse has been generated, with MS2 labelling of the β-actin gene, as proof of the principle that RNA tagging is nonlethal (101). A current disadvantage of this model is that the MS2 bacteriophage coat protein is not endogenously expressed limiting analyses to *ex vivo* cells amenable to transfection techniques, thus concomitantly decreasing similarities to endogenous cells within tissue environments. By contrast, a transgenic rat model enabling indirect measurement of transcription activity through reporter gene expression allows measurement of real-time transcription activity in cells in *ex vivo* tissue preparations and primary cultures. This system is being exploited to assess how tissue environment, tissue development and changes in tissue physiology affect transcription activity (15,102,103).

Currently, it is not possible to measure the dynamics of cellular processes, such as transcription, in single cells, *in vivo*, in whole animals, where multiple organ systems interact to maintain normal physiology. Although technologies such as optical imaging exist, which can detect fluorescent or luminescent labels *in vivo*, these technologies have limited spatial resolution and limited penetrance through animal tissues. Developments are already being made to combat these issues (e.g. via the use of fibre optic probes). Future developments may aim to increase the sensitivity of these systems and to make them implantable into animal tissues to enable long-term imaging studies.

Indirect measurement of transcription activity in tissue *ex vivo* using a transgenic rat model was developed using the human prolactin locus as a model of complex gene regulation. Reporter genes (luciferase and destabilised GFP) were knocked-in to the first coding exon of the human prolactin locus encoded in a BAC, which enables appropriate regulation of the transgene (Fig. 3b). The position of the reporter genes prevents translation of the prolactin protein from the transgene with the maintenance of normal prolactin levels from endogenous gene expression. A comparison of dispersed cells in primary culture with cells maintained within intact tissue shows clear differences in prolactin transcription profiles in both the adult and developing pituitary (Fig. 3b) (102,103), indicating that pituitary tissue environment and cellular communication have an impact on lactotroph transcription activity. Cellular organisation in pituitary tissue has been suggested to be important for other cellular functions; for example, the coordinated secretion of hormone from somatotroph cells (104) and functional connectivity of lactotroph cells through gap junction signalling (105).

In adult pituitary tissue, adjacent cells show heterogeneous transcription patterns but the summation of these patterns enables coordinated behaviour across the tissue (102). It is unclear whether the heterogeneous transcription patterns result from functional heterogeneity of the cells, differences in cell cycle stage and/or the intrinsic factors noted above, such as differences in oscillatory protein activity, chromatin cycle and transcription cycle stage. The heterogeneity in transcriptional activity in the tissue may facilitate graded responses to transient and sustained stimulation by affecting not only the transcription rate within individual cells, but also the number of cells recruited to the expressing cell population. It should be noted that lactotroph cells have heterogeneous protein storage characteristics (106) and that a direct relationship between gene transcription and protein secretion is unlikely, given that there are other downstream influences such as post-transcriptional regulation, mRNA degradation rate, translation rate, protein storage and regulated secretion. The significance of processes occurring downstream of transcription on prolactin protein secretion dynamics are suggested by a lack of correlation between prolactin transcription rate, mRNA accumulation and protein secretion (80,107).

In pituitary development, the transcription patterns of the prolactin gene are altered between nascent lactotroph cells in the embryonic pituitary and more mature cells in neonatal pituitaries (Fig. 3b). This suggests that the fundamental factors that determine transcriptional pulsatility, such as chromatin cycles, are variable (103). The factors that facilitate changes in transcription pulsatility during development (yet to be defined) may provide essential clues as to the key controllers of transcriptional pulsatility. Furthermore, early prolactin expression in newly-derived lactotroph cells occurred in highly transient pulses, which may have implications for the programming of gene expression in differentiating cells. Questions remain as to whether and how transcription patterns change in response to physiological demand (e.g. during lactation and in response to circadian rhythms) and how tissue architecture may affect transcription activity.

## Chromatin structure of the prolactin gene and potential influences on transcription dynamics

The major influence of chromatin on nuclear processes, such as transcription and replication is well established and, as mentioned above, chromatin has a large affect on the transcription dynamics of mammalian genes, including the prolactin gene. Chromatin is the complex of DNA and all associated protein and RNA and is mainly comprised of histone proteins. H2A, H2B, H3 and H4 form the nucleosome around which DNA is wrapped, and H1, the linker histone, binds in between nucleosomes aiding folding and chromatin stabilisation. Modification of chromatin structure occurs through the post-translational modification of histones, the substitution of canonical histones with histone variants and by remodelling of nucleosome position by ATP-dependent chromatin remodelling proteins (108). Chromatin regulation of the human prolactin locus has not been studied in great detail as a result of technical limitations, particularly the lack of a cellular model. Although it is known that chromatin structure has an influence on human prolactin transcription, as revealed by histone deacetylase inhibition by Trichostatin A (87), chromatin structure across the locus and trans-acting factors and mechanisms responsible for its remodelling remain undetermined. In the rat prolactin locus, the chromatin structure of the locus and its remodelling have been more fully investigated, although further characterisation is required for a complete understanding of chromatin influence on prolactin expression.

In general, the kinetics of chromatin remodelling and correlation with transcriptional activity are relatively unknown. Inducible transcription reporter systems have been used to assess changes to chromatin with the onset of transcription (109). However, changes in chromatin structure and function and correlation to pulsatile transcription at endogenous genes have not yet been determined. At the prolactin locus, real-time imaging of a prolactin enhancer/promoter array, visualised by GFP–ER interactions, showed large-scale chromatin decondensation and recondensation with differing temporal dynamics upon induction and removal of epidermal growth factor (EGF) and oestrogen. Interestingly, transcription dynamics correlated with the chromatin state for EGF but not for oestrogen, which induced pulses of gene expression (94). The relation of this visual assessment of chromatin state (large-scale) to more precise changes in chromatin structure (e.g. histone modifications and chromatin remodelling of nucleosome position) is unknown and awaits technical advances that will allow chromatin analyses on single cells (or at least synchronised cell populations).

### Nucleosomal influences on transcription dynamics

Nucleosome positioning may affect transcription dynamics through competition with transcription factors for DNA binding sites. The positioning of nucleosomes has a large impact on transcription activity, with remodelling being required to expose transcription factor and RNA polymerase-binding sites in the promoter and also to allow RNA polymerase to transit through the gene. Nucleosome positioning is defined through sequence preferences and competition with other DNA binding proteins; thus, nucleosome positioning is plastic, reflecting equilibrium with DNA binding protein concentrations. This is demonstrated in genome-wide mapping studies of nucleosome position, which report that approximately 80% of the genome shows no preferential positioning of nucleosomes (110). Stochasticity in gene expression may therefore be affected by transcription factor concentration and DNA binding site affinity, such that low levels of gene expression noise would be predicted at nucleosome disfavouring sequences, whereas low concentrations of transcription factors or low affinity binding sites would facilitate highly variable levels of gene expression (Fig. 4b) (111,112). At regulatory regions of the prolactin locus, nucleosome positioning is altered between expressed and non-expressed states, with analyses of mini-chromosomes suggesting that nucleosomes have defined positions in expressed states but are positioned randomly in non-expressed states (113,114). Pit-1 has been shown to alter nucleosome positioning on prolactin constructs reconstituted into chromatin *in vivo*, independently from its function in transcriptional activation (115). These data suggest that prolactin transcription dynamics may be influenced by competition between nucleosomes and Pit-1, and potentially other factors, for DNA binding. However, in mammalian cells, reporter systems show that increasing the levels of transcription factor or the number of binding sites influences the transcription burst size but has little effect on transcription burst frequency, indicating that transcription dynamics are not solely dependent on transcription factor binding kinetics (75).

Nucleosome positioning may also be affected by Z-DNA. Z-DNA formation is favoured by alternating purine–pyrimidine sequence repeats, generating a nonclassical DNA structure with a left-handed helix. The biological role of Z-DNA has not been fully elucidated, although it may have roles in transcription initiation and chromatin remodelling (116). There is evidence that Z-DNA may prevent nucleosome binding to DNA following nucleosome eviction by chromatin remodelling enzymes, facilitating the interaction of the transcription machinery with promoter sequences (117,118). In the rat prolactin locus, Z-DNA has been detected flanking the distal enhancer, with the downstream Z-DNA sequence having a negative influence on prolactin expression (119,120). In the human prolactin locus, the Z-DNA sequences have been reduced to a single shorter sequence downstream of the distal enhancer. Interestingly, an extensive Z-DNA repeat is located in intron 4–5 of the human and rat prolactin loci, although the functional role of this is unknown.

### Histone post-translational modification at the prolactin gene locus

The structure and function of chromatin is also modulated by the post-translational modification of histone proteins by phosphorylation, acetylation, methylation, ubiquitination, SUMOylation, ADP ribosylation, deimination and proline isomerisation. Modification of histones affects the interaction of the DNA with the nucleosome and also generates binding motifs enabling the recruitment of regulatory proteins. The modification of histones, along with protein constituents, is grouped to define a small number of chromatin domains that can be linked to gene activity (108). The modification of histones is not permanent, with histone turn over occurring similarly to cell cycle timing. Histones with modifications associated with active transcription have been shown to turn over faster than histones with silent modifications (121), and histone-modifying enzymes (histone acetyltransferases and histone descetylases) have been associated with active genes, enabling a resetting of chromatin structure following transcription (122). The relationship of these activities to transcription dynamics remains to be determined.

At the prolactin locus, Pit-1 and ER may be able to alter chromatin structure by histone modification via the recruitment of co-repressor and co-activator complexes and remodelling enzymes (123–125). Chromatin remodelling at the prolactin locus is not equivalent between different transcriptional regulators of the gene. Oestrogen and EGF cause chromatin decondensation over differing time scales (94) and, in contrast to dopamine and oestrogen, which alter H4 acetylation levels, dexamethasone has no affect on H4 acetylation (124). Chromatin remodelling facilitated by Pit-1 also potentially differs depending upon the intracellular signalling pathways used as a result of the recruitment of different histone acetyltransferase proteins (123) and through the use of different Pit-1 isoforms that differ in their ability to modify histone proteins (126). These data suggest that not all signalling pathways regulate the prolactin locus through the same chromatin remodelling pathway and that numerous chromatin remodelling proteins may be employed to regulate the gene. Thus, it is possible that distinct histone modifications, induced by different signalling pathways, may generate an epigenetic memory at the prolactin locus, although this requires further investigation. Overall, it is clear that chromatin has a large impact upon transcription; however, the affect individual components have on the dynamics of transcription requires further detailed investigations.

## Perspective

Despite the wealth of data on the regulation of the prolactin gene, it is still unclear how cell-specific activity is conferred on the prolactin gene in both pituitary and extra-pituitary sites and a full understanding of how regulation is conferred upon the gene by numerous proteins along with the spatial organisation of the locus within the nucleus is incomplete. Emerging technologies will hopefully provide the resources needed to shed light on these unresolved issues. In particular, the increasing tractability of BAC transgenesis (14) will enable the use of larger constructs that will help to address how regulatory elements integrate their activity. Pulsatile expression patterns of the prolactin gene, probably alterable by tissue structure, also suggests the complex regulation of transcription activity and cell function. Technological advances in the detection of RNA, through the use of different reporter systems and increasingly sophisticated microscope technology, will also enable further investigations of the real-time activity of the prolactin locus in single cells, providing essential clues about the activity of the gene in physiological and pathological states.
